# Death Related to a Congenital Vascular Anomaly of Pulmonary Hamartoma Type: Malpractice or Tragic Fatality?

**DOI:** 10.3390/medicina57111231

**Published:** 2021-11-11

**Authors:** Maricla Marrone, Laura Marrone, Gerardo Cazzato, Stefania Lonero Baldassarra, Giuseppe Ingravallo, Alessandra Stellacci

**Affiliations:** 1 Legal Medicine Section, Interdisciplinary Department of Medicine, University of Bari “Aldo Moro”, 70124 Bari, Italy; mariclamarrone@hotmail.it (M.M.); stefania.lonerobaldassarra@uniba.it (S.L.B.); alestellacci@gmail.com (A.S.); 2 Military Court Judge of Verona, 37100 Verona, Italy; rosauramarrone@gmail.com; 3 Pathology Section, Department of Emergency and Organ Transplantation DETO, University of Bari “Aldo Moro”, 70124 Bari, Italy; giuseppe.ingravallo@uniba.it

**Keywords:** medical malpractice, defensive medicine, bronchoscopy, doctor-patient relationship, autopsy, COVID-19

## Abstract

In forensic pathology, apparently straightforward cases can often hide rarities that, if not correctly interpreted, can alter the results of the entire investigation, leading to misinterpretations. This occurs when the investigation is conducted to assess medical malpractice. An unexpected death, with no known apparent cause, is often linked to an underlying disease process of unclear etiological origin whose nature can, unfortunately, be properly investigated only post-mortem. This presentation shows a case study, in which it was possible to reconduct the death of a patient to a natural pathology and not to medical treatment. Here, the authors illustrate a case with a hamartoma developed in chronic inflammatory conditions (bronchiectasis) that was difficult to differentiate from lung cancer due to the inability to perform specific instrumental examinations. The hamartoma, usually benign and identifiable by standard instrumental investigations, in this case, led to the patient’s death precisely during the execution of a bronchoscopy. However, in the absence of a certain cause of death, public opinion unanimously attributes a patient’s disease to medical error. Indeed, a routine practice such as bronchoscopy should not cause death and consequently, the doctor must have made a mistake. Fortunately, the autopsy not only demonstrated the origin of the bleeding but also unveiled the reason for this, as rare congenital lung disease. Fate, one might say.

## 1. Introduction

In forensic pathology, apparently straightforward cases can often hide rarities that, if not correctly interpreted, can alter the results of the entire investigation, leading to misinterpretations. This occurs, in particular, when the investigation is conducted to assess medical malpractice. In these circumstances, apart from the normal individual variables, the fatal outcome of a medical procedure (wrong choice of treatment or unsuitable clinical/surgical practice) may be correlated, rather than with medical negligence, with rare diseases that are difficult to diagnose, little studied and hard to recognize, and whose evolution may be difficult to predict [1,2]. An unexpected death, with no known apparent cause, is often linked to an underlying disease process of unclear etiological origin whose nature can, unfortunately, be properly investigated only post-mortem. In the field of medical responsibility, forensic medicine imposes as a prerequisite the existence of several causal criteria for the recognition of a possible health error. The chronological criterion alone is not enough, which, unfortunately, is sufficient to guide public opinion [3].

Suffice it to think, in the present, how the figure of the doctor in the era of COVID-19, from an initial exaltation to hero, has become the subject of complaints, following the occurrence of deaths. Deepening, therefore, through the autopsy of medical-legal investigation of “doubtful” cases. Moreover, in the context of a pandemic, it promotes the scientific understanding of the events, but can also help in restoring confidence to health care. Here, the authors illustrate a case with a hamartoma developed in chronic inflammatory conditions (bronchiectasis) that was difficult to differentiate from lung cancer due to the inability to perform specific instrumental examinations.

## 2. Case Presentation

The authors report the case of a 49-year-old man with anatomical-dysfunctional abnormalities in the lungs (a small, irregularly shaped right lung and bronchiectasis), who underwent fibrobronchoscopy at the Hospital in the South of Italy to define the nature of a mass. In this case, the mass was already identified by chest X-ray examination and confirmed by a thorough computerized tomography (CT) examination. During the examination, especially after introducing the tool and having aspirated a large amount of thick mucus from the tracheobronchial level in an attempt to make a second bronchial aspiration, massive bleeding occurred which dictated the infusion of cold distilled water to induce vessel constriction. The endoscopic maneuver was interrupted [4,5]. Shortly afterward, despite all of the resuscitation and pharmacological measures performed, the patient died of cardiopulmonary arrest secondary to hypovolemic shock and internal drowning.

The autopsy was required by the magistrate to evaluate whether the doctor who had performed the bronchoscopy was liable for malpractice. It was assumed that a wrong introduction of the endoscopic instrument in the airways might have damaged a part of a large vessel and that the copious hemorrhage was consequent to an inadvisable biopsy. The preliminary examination of the corpse excluded any sign of external traumatic injury. In addition, the examination showed that abundant blood contaminated the clothes worn by the victim. Moreover, when handling the corpse, the leakage of bright red blood from both nostrils was shown.

**Figure 1:**
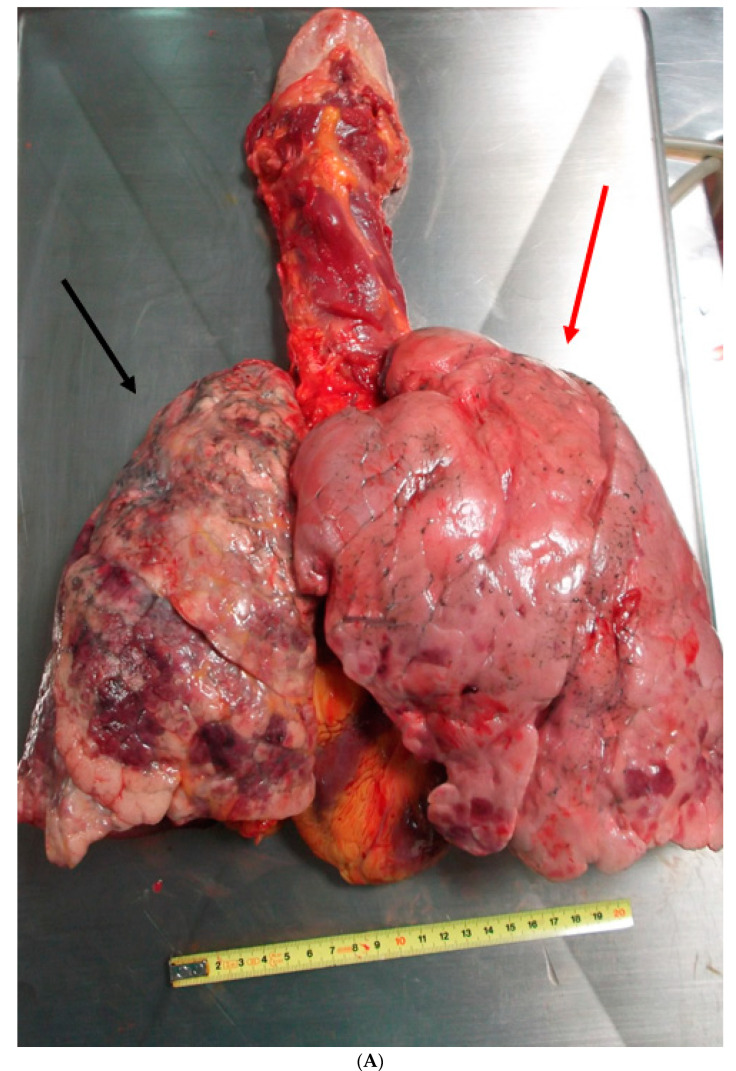

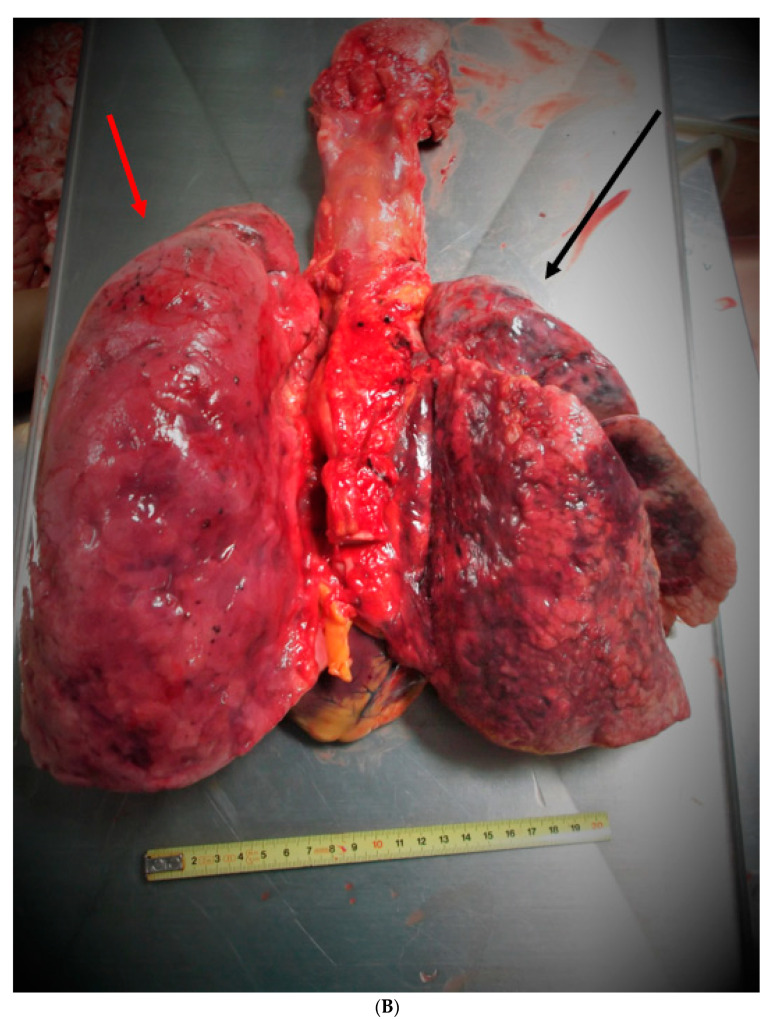
(**A**) Anterior view. Heart-lung block showing the right lung strongly retracted, with discolored and petechial hemorrhagic areas on the outer surface (black arrow). Left lung without evident macroscopic alterations (red arrow). (**B**) Posterior view. Heart-lung block showing the right lung strongly retracted, with discolored and petechial hemorrhagic areas on the outer surface (black arrow). The left lung does not present particular macroscopic alterations (red arrow).

**Figure 2:**
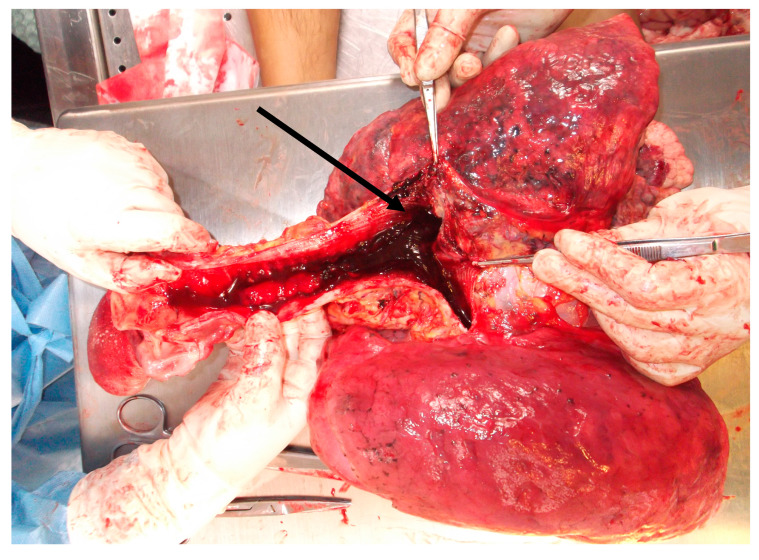
Posterior view. The trachea with its bifurcations entirely occupied by blood material both in the larger diameter ducts and in the smaller caliber airways (black arrow).

**Figure 3:**
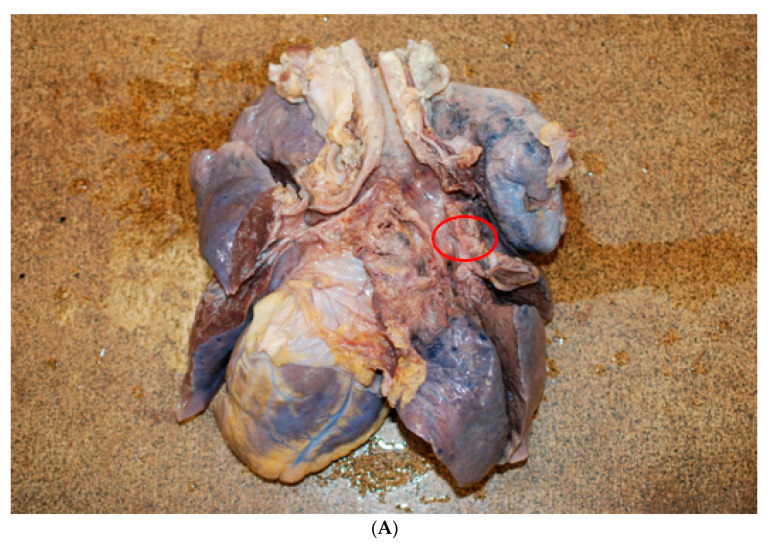

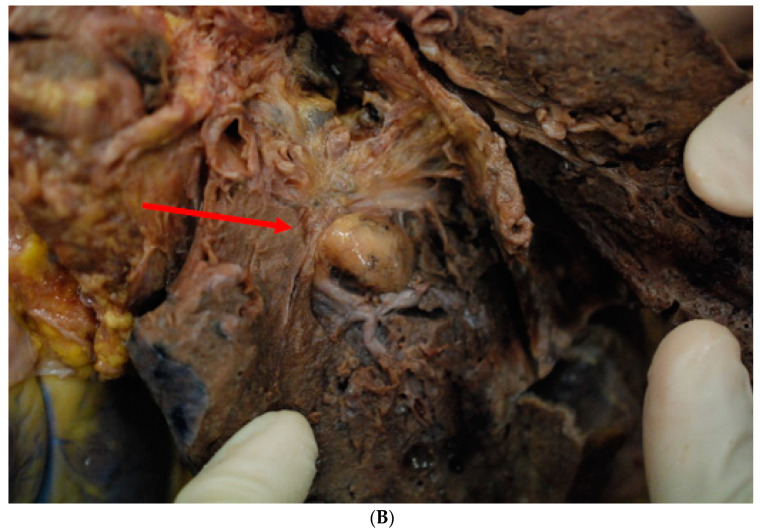
(**A**) The external surface between the superior and medial right bronchus, a mass, not protruding into the lumen, but with a rich vascularization that was partially fused to the bronchial wall, which appeared thinned and delaminated (red circle). (**B**) The arteriovenous malformation of the hamartomatous type after fixation of the sample in 10% neutral buffered formaldehyde (red arrow).

A further examination of the cadaver revealed myocardial hypertrophy and a marked anatomical alteration of the right lung (weight 450 g, length 24 cm, and diameter at the base of 15.5 cm), that showed a much smaller size and volume than the left lung (550 g, length 21.5 cm, and diameter at the base of 13 cm). The entire parenchyma had a greater consistency (Figure 1A,B). The trachea and bronchi showed the presence of many blood clots bilaterally (Figure 2). Moreover, in the initial tract of the right bronchus, immediately after the bifurcation, a dense, congested capillary network was evident on the endoluminal surface. In addition, on the external surface between the superior and medial right bronchus, a mass could be seen, not protruding into the lumen but with a rich vascularization that was partially fused to the bronchial wall, which appeared thinned and delaminated. In particular, an accurate macroscopic examination of the respiratory tract showed a lobulated mass involving the trachea from the carena to the right bronchus and the middle lobe of the right lung, which was decreased in weight. The mass was soft and bluish-grey, spongy on the cut surface, and it was not resectable from the tracheobronchial wall (Figure 3A,B).

**Figure 4:**
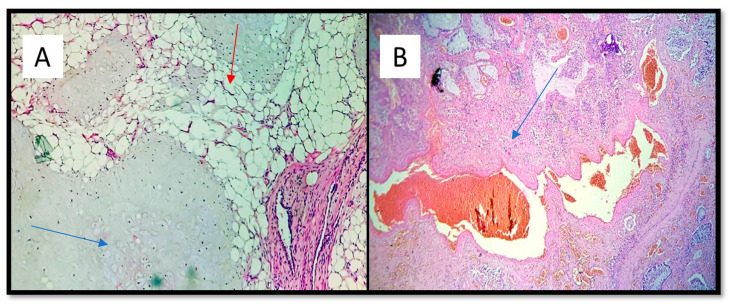
(**A**) Three components of pulmonary hamartoma: Cartilage (blue arrow), adipose tissue (red arrow), and vascular tissue (hematoxylin-eosin, original magnification: 10×). (**B**) Abnormal presence of arterial and venous vascular tissue. Note the presence of thrombus and chronic flogistic infiltration outbreaks (blue arrow) (hematoxylin-eosin, original magnification 10×).

**Figure 5:**
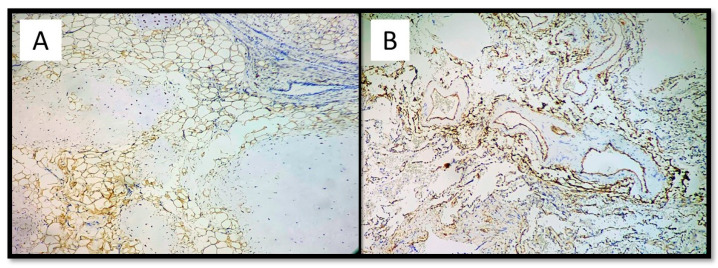
(**A**) Immunostaining for vimentin which shows positivity in the adipose component and negativity in the vascular and cartilage component of the hamartoma (immunohistochemistry, original magnification: 10×). (**B**) Immunostaining for CD34 showing extensive and widespread positivity in the vascular component of the hamartoma (immunohistrochemistry, original magnification: 10×).

An histological examination demonstrated that the mass consisted of a congenital vascular anomaly of hamartoma type [6]. Moreover, the histological examination ruled out any tears of this congenital malformation caused by the biopsy instrument. Microscopically, the lesion resembled capillary or cavernous angiomas and some dilated vessels had a smooth muscular wall. These abnormal vessels were lined with flattened endothelial cells and their structures varied from arteries to venous in different areas, sometimes filled with thrombi beginning to aggregate. No other co-existing vascular anomalies were noted in other pulmonary lobes and organs. The finding was suggestive of an arteriovenous malformation of the lung (maybe congenital). Histologically, the right pulmonary parenchyma showed peripheral emphysematous areas alternating with fibro-atelectatic areas. The interstitial connective tissue was densely infiltrated by lymphohistiocytic inflammatory cells (Figure 4). Immunohistochemical investigations showed positivity for Vimentin in the mesenchymal component (adipose tissue), while it was negative in the cartilage component (Figure 5A). CD34 positivity highlighted the vascular malformative component (Figure 5B).

## 3. Results

Despite these macro and microscopic findings, doubts remained about the work of the health professionals who were responsible for the patient: Was the presence of a richly vascularized formation enough to contraindicate a fibrobronchoscopic examination with biopsy? Why was it performed? The answer to this question was provided by a careful examination of the health records. In reality, neither the chest X-ray examination nor the CT scan could distinguish the vascular nature of the lesion. A CT scan with a contrast medium would have been necessary, but although initially recommended, it could not be performed since the patient had severe chronic kidney disease (stage IV) and could not tolerate the administration of the appropriate contrast medium. At this point, the only possibility to define the nature of the lung mass was, according to the guidelines, to perform fibrobronchoscopy. Therefore, this was performed in this case. At the present time, although this procedure was conducted according to lege artis, the tangle of vessels broke and triggered the lethal hemorrhage. This prevented biopsy sampling.

The histological examination showed the absence of iatrogenic injury to load both of the hamartoma of the surrounding tissues. Bronchoscopy had not affected the tumor exactly as described in the medical report. The rare findings, in this case, confirmed that the copious bleeding was a consequence of the laceration caused by the pressure induced by insertion of the bronchoscope in the bronchial tree, owing to its intrinsic morphologic-structural characteristics (tortuous vessels content, thin aneurysmatic walls).

In particular, the intrinsic morphologic-structural characteristics of the bronchial malformation solicited by a hypertensive state baseline (in part induced by renal failure and myocardial hypertrophy), the vasal fragility resulting from kidney failure and hypertension caused by the agitation of the patient (described in documents), and the resulting pressure jump aspiration of the mucus, determined the lethal hemorrhage.

## 4. Discussion

Direct communications between the branches of the pulmonary artery and pulmonary veins, without an intervening pulmonary bed, are probably the most common anomalies of the pulmonary vascular tree. In addition, they have been variously called a pulmonary arteriovenous fistula, pulmonary arteriovenous malformation (PAVM), pulmonary arteriovenous aneurysm (PAVA), pulmonary angioma, arteriovenous angiomatosis, cavernous haemangiomas, and pulmonary hamartomas [7,8,9,10,11,12,13]. The lesions usually represent congenital malformation, except for very rare acquired cases. They are an important part of the differential diagnosis of common pulmonary problems such as hypoxemia and pulmonary nodules [14,15]. Communications between bronchial arteries and pulmonary arteries, causing the left-to-right shunt, can develop in chronic inflammatory conditions such as bronchiectasis.

In particular, hamartomas are tumor-like malformations due to the abnormal mixing or abnormal development of normal tissue components of the organ in which they occur. This abnormality is thought to be the result of variations in the quality, arrangement or degree of differentiation of the tissues. Hamartoma has a high frequency accounting for 77% of all the benign lung tumors. Often discovered incidentally, they are typically well-circumscribed nodules or masses (usually small) with smooth or lobulated margins. Approximately 60% have fat [16] and approximately 20–30% have calcification/ossification (popcorn-like). In addition, cavitation is not seen [13,16,17]. The majority of pulmonary hamartomas are asymptomatic and show slow annual growth. However, it is also important to recognize that some hamartomas might increase rapidly in size and show malignant alteration [18]. Growth occurs but is very slow, with a typical volume doubling time (VDT) of over 400 days. As for the radiographic characteristics, they are typically smooth, well-circumscribed nodules, round or oval, with heterogeneous internal structures. Moreover, popcorn-like calcification may be observed [19]. The CT-scan of vascular tree is useful for the differentiation of pulmonary mass lesions. In our case, the doctors could not perform an angio-tac (due to the kidney disease). In addition, it was impossible to identify numerous vessels in the mass. Moreover, fluorodeoxyglucose (FDG) positron emission tomography (PET) is useful for the differentiation of pulmonary mass lesions [20]. However, some reports on hamartomas describe the difficulty with differentiation from malignant tumors when a slight abnormal accumulation of FDG is recognized. Hamartoma is considered to be a benign tumor with a good prognosis and is often followed by an observation without surgery due to the slow annual growth. However, rapid growth or a high concentration of vessels especially in people with high blood pressure or chronic diseases can have serious consequences on the health of the patient. In any case, these injuries after proper identification, should be reviewed regularly in the patient and his family must be evaluated for possible similar injuries. Hamartomas can be characteristic of a syndrome, known as phosphatase and tensin homolog (PTEN) hamartoma tumor syndrome (PHTS). This syndrome encompasses four major clinically distinct syndromes associated with germline mutations in the tumor suppressor PTEN phosphatase and tensin homologue, situated on chromosome 10, involved in DNA repair, cellular senescences, cell migration/metastasis, stem cell self-renewal, and maintaining genomic stability [21]. These allelic disorders, Cowden syndrome, Bannayan-Riley-Ruvalcaba syndrome, Proteus syndrome, and Proteus-like syndrome are associated with unregulated cellular proliferation leading to the formation of hamartomas. To date, an increased risk of malignancy has only been documented in Cowden syndrome. However, current recommendations advise that all of the individuals with PTEN hamartoma tumor syndrome follow the cancer surveillance strategies suggested for Cowden syndrome until further data indicate otherwise [22].

The presence of mutations in the PTEN gene increases the risk of developing benign and malignant tumors in many other districts [23]. It would be desirable to direct the patient’s family to genetic counseling, for the identification of mutations of the PTEN gene involved in the pathogenesis of the disease. In addition, for conducting research on the affected individuals and healthy families for an early diagnosis and monitoring of the disease.

There are several cases of hamartomas in the literature, not only in the lungs. A few of them were identified by CT or FDG-PET [22], while others were identified with bronchoscopy [24].

In the work of the doctors involved in the case, there was nothing wrong. Therefore, the decision to deepen the diagnostic investigation was correct, as it was not possible to determine in advance that it was a rare malformation.

However, in the absence of a certain cause of death, public opinion unanimously attributes a patient’s decease to medical error [25,26,27]. Indeed, a routine practice such as bronchoscopy should not cause death and consequently, the doctor must have made a mistake. Fortunately, the autopsy not only demonstrated the origin of the bleeding, but also unveiled the reason for this, as a rare congenital lung disease. Fate, one might say.

There is an increasing tendency to blame the doctor for medical failures. If this is not restrained, it will lead to unwarranted “defensive” medicine, ultimately harming patients, above all [28,29,30,31]. Moreover, the pressure on doctors is evident due to the fact that the cost of medical insurance is rising at an increasing rate, owing to the decline of responsibility of the State health system [32]. This entails not a lack of adequate training of young doctors or adequate updating by seniors, but surely a deficient quality of the doctor-patient relationship and poor work conditions. These are the factors that lead to increased lawsuits.

## 5. Conclusions

The improved living conditions and the models shared by media and advertising are the cause of an exasperated “witch hunt”, unfortunately, related only to economic causes based on the inauspicious principle that “someone must pay” and not on real health needs.

## Data Availability

Not applicable.
